# Exposure history, post-exposure prophylaxis use, and clinical characteristics of human rabies cases in China, 2006–2012

**DOI:** 10.1038/s41598-018-35158-0

**Published:** 2018-11-21

**Authors:** Chun Guo, Yu Li, Yang Huai, Carol Y. Rao, Shengjie Lai, Di Mu, Wenwu Yin, Hongjie Yu, Shaofa Nie

**Affiliations:** 1 Department of Epidemiology and Biostatistics, School of Public Health, Tongji Medical College, Huazhong University of Science and Technology, Wuhan, Hubei China; 2 Key Laboratory of Surveillance and Early-warning on Infectious Disease, Division of Infectious Disease, Chinese Center for Disease Control and Prevention, Beijing, China; 3 WHO Collaborating Centre for Infectious Disease Epidemiology and Control, School of Public Health, The University of Hong Kong, Hong Kong, China; 4 International Emerging Infections Program, Division of Global Health Protection, Centers for Disease Control and Prevention, Beijing, China; 5 Division of Global Health Protection, Centers for Disease Control and Prevention, Atlanta, Georgia USA; 6 WorldPop, Department of Geography and Environment, University of Southampton, Southampton, UK; 7 School of Public Health, Fudan University, Key Laboratory of Public Health Safety, Ministry of Education, Shanghai, China

## Abstract

Rabies is still a public health threat in China. Evaluating the exposure history, clinical characteristics, and post-exposure prophylaxis (PEP) of the cases could help in identifying approaches to reducing the number of these preventable deaths. We analysed data collected from 10,971 case-investigations conducted in China from 2006 to 2012. Most cases (n = 7,947; 92.0%) were caused by animal bites; 5,800 (55.8%) and 2,974 (28.6%) exposures were from domestic and free-roaming dogs, respectively. Only 278 (4.8%) of these domestic dogs had previously received rabies vaccination. Among all cases, 5,927 (59.7%) cases had category III wounds, 1,187 (11.7%) cases initiated the rabies PEP vaccination and 234 (3.9%) cases with category III wounds received rabies immunoglobulin. In our adjusted logistic regression model, male cases (adjusted odds ratio [aOR] = 1.25, 95% confidence interval [CI]: 1.09–1.44) and farmers (aOR = 1.39, 95% CI: 1.10–1.77) and person older than 55 years (aOR = 1.48, 95% CI: 1.01–2.17) were less likely than females and persons in other occupations or younger than 15 years to initiate PEP vaccination. The median incubation period was 66 days (interquartile range (IQR): 33–167 days). To reduce the number of human deaths due to rabies, rabies prevention campaigns targeting males and farmers and older people should be conducted. Increasing routine rabies vaccination among domestic dogs will be essential in the long term.

## Introduction

Rabies is an acute and fatal zoonotic disease commonly transmitted to humans through a bite or scratch from an infected animal^1^. Outbreaks of rabies, which can result from uncontrolled populations of rabid animals, represent a health security threat. Globally, rabies causes approximately 59,000 human deaths every year, 95% of which occur in Asia and Africa^2,3^. Progression to infection after exposure to rabies can be prevented with post-exposure prophylaxis (PEP), comprising of appropriate wound treatment, followed by completion of the rabies PEP vaccination series and the administration of rabies immunoglobulin (RIG) when warranted^4^. Despite these effective treatments, between 1960 and 2014, there have been an average of 2,198 rabies-related deaths each year in China, and so rabies remains a considerable public health threat^5^.

Rabies has been a notifiable disease in China since 1955^5^, with case reporting and investigation implemented in 2005. Medical institutions must report all clinically diagnosed and laboratory-confirmed rabies cases to the National Notifiable Infectious Disease Reporting Information System (NIDRIS), after which, county-level Centers for Disease Control and Prevention (CDC) initiate case investigations. China’s national policy requires wound treatment and PEP vaccination for category II and category III exposures, as well as RIG administration for category III exposure^6^. The PEP vaccination series can be administered as either Zagreb 2–1–1, in which two doses of vaccine are injected intramuscularly on day 0 (one into each of the two deltoid or thigh sites) followed by a single dose on days 7 and 21, or the five-dose Essen regimen, in which one dose is administered intramuscularly on days 0, 3, 7, 14, and 28, based on the World Health Organization (*WHO) Expert Consultation on Rabies (2013 version)*^4^. All patients with exposure categories II or III should initiate either series as soon as possible following possible rabies exposure^4^. At present, human rabies immune globulin (HRIG) and equine rabies antiserum (ERA) are approved for use in China, however, patients typically pay for the PEP vaccination series and RIG as ‘out-of-pocket’ expenses.

Rabies vaccination coverage for dogs remains low in China^7^. Although there are numerous free-roaming dogs in China, no national programme exists for mandatory rabies vaccination. As a result, the prevention of human rabies relies on community rabies awareness, as well as access to health care for appropriate administration of PEP vaccinations and RIG as recommended by national policy. In this project, we analysed information from human rabies case investigations, conducted between 2006 and 2012, in order to describe exposure history, clinical characteristics and PEP practices of rabies cases in China. Our findings can help to target future interventions, and to improve community awareness and clinical practice involving rabies exposures.

## Results

### Characteristics of cases and rabies exposures

We obtained data from 11,902 case investigations, performed between January 1 2006 and December 31 2012, from 30 of the 31 provinces in China (no rabies case were reported from Tibet during the project period^8^). We then cross-referenced these case investigations with the 16,628 rabies cases reported to NIDRIS for the same period. Of the case investigations, 10,971 were successfully matched to NIDRIS. Unmatched case investigations included 931 suspected rabies cases (did not conform to the clinically diagnosed or laboratory-confirmed case definitions), which are not reportable to NIDRIS. Since investigation of human rabies cases (data included in this analysis) is not mandatory but only encouraged, we did not receive data for the remaining 5,657 (34%) clinically diagnosed or laboratory-confirmed cases reported to NIDRIS.

Of the 10,971 successfully matched cases, 10,818 (98.6%) were diagnosed clinically and 153 (1.4%) were diagnosed by laboratory testing. Most rabies cases were male (n = 7,615; 69.4%), farmers (n = 7,624; 71.8%), and lived in rural areas (n = 7,443; 76.4%). Cases were primarily aged 55–64 years (n = 2,290; 20.9%) followed by those ≥65 years (n = 2,062; 18.8%) and <15 years (n = 1,980; 18.0%).

The majority (n = 7,947; 92.0%) of human rabies cases were associated with an animal bite (Table 1). Exposures were most frequently caused by the case’s own domesticated dog (n = 3,875; 37.3%), a free-roaming dog (n = 2,974; 28.6%), or a neighbor’s domesticated dog (n = 1,925; 18.5%). Wounds from domesticated animals (dogs and cats) were responsible for more than half (n = 6,143; 59.1%) of the investigated rabies cases. Of these domesticated animals, only 292 (4.8%) were vaccinated against rabies (278 dogs and 14 cats). One suspected person-to-person transmission was reported, in which a 54-year-old mother was infected with rabies after being bitten by her infected son. Due to the lack of laboratory testing, however, this person-to-person transmission could not be confirmed.

**Table 1:** Characteristics of exposure history and post-exposure prophylaxis, according to data from human rabies case investigations, China, 2006–2012.

Characteristics	Diagnosis type, n (%)		Total, n (%)
	Clinically diagnosed cases	Laboratory-confirmed cases	
Exposure route			
Bite	7,828 (92.0)	119 (93.7)	7,947 (92.0)
Scratch	681 (8.0)	8 (6.3)	689 (8.0)
Total	8,509 (100.0)	127 (100.0)	8,636 (100.0)
Attacking animal			
Domesticated dog from own home	3,838 (37.4)	37 (26.2)	3,875 (37.3)
Free roaming dog	2,930 (28.6)	44 (31.2)	2,974 (28.6)
Domesticated dog from neighborhood	1,893 (18.5)	32 (22.7)	1,925 (18.5)
Domesticated cat from own home	272 (2.6)	8 (5.7)	280 (2.7)
Wild animal^a^	192 (1.9)	3 (2.1)	195 (1.9)
Free roaming cat	86 (0.8)	0 (0.0)	86 (0.8)
Domesticated cat from neighborhood	62 (0.6)	1 (0.7)	63 (0.6)
Other animal^b^	981(9.6)	16 (11.4)	997 (9.6)
Total	10,254 (100.0)	141 (100.0)	10,395 (100.0)
Exposure category			
II	2,684 (27.4)	41 (30.2)	2,725 (27.4)
III	5,846 (59.7)	81 (59.6)	5,927 (59.7)
II or III^c^	720 (7.3)	7 (5.1)	727 (7.3)
Unknown^d^	547 (5.6)	7 (5.1)	554 (5.6)
Total	9,797 (100.0)	136 (100.0)	9,933 (100.0)
Type of wound treatment			
Only treated by oneself	2,697 (24.9)	49 (32.0)	2,746 (25.0)
Only treated in medical institution	1,040 (9.6)	13 (8.5)	1,053 (9.6)
Both of above	42 (0.4)	0 (0.0)	42 (0.4)
No treatment	7,039 (65.1)	91 (59.5)	7,130 (65.0)
Total	10,818 (100.0)	153(100.0)	10,971 (100.0)
Receive PEP vaccination^e^			
Yes^f^	1,174 (11.7)	13 (9.5)	1,187 (11.7)
No	8,854 (88.3)	124 (90.5)	8,978 (88.3)
Total	10,028 (100.0)	137 (100.0)	10,165 (100.0)
Injecting RIG or not			
Yes	278 (3.0)	2 (1.5)	280 (3.0)
No	8,868 (97.0)	132 (98.5)	9,000 (97.0)
Total	9,146 (100.0)	134 (100.0)	9,280 (100.0)

Note: ^a^Wild animals included rats (n = 14), masked palm civets (n = 2), skunks (n = 2), wolves (n = 1), bat (n = 1), marmots (n = 1), badgers (n = 1), otters (n = 1), squirrels (n = 1), wild boars (n = 1).

^b^Other animals included rats from own home (n = 3), rats from unknown place (n = 7), stray rat (n = 1), pigs from own home (n = 10), pigs from neighborhood (n = 2), pigs from pig farm (n = 1), horses from own home (n = 2), horses from unknown place (n = 1), donkeys from own home (n = 1), cattle from own home (n = 1), cattle from unknown place (n = 1), rabbits from own home (n = 2), foxes from own home (n = 1), raccoon dogs from own home (n = 1).

^c^Exposure caused by animal bite or scratch but classified as category I by medical staff.

^d^Exposure classified as category I by medical staff, but exposure route was unknown.

^e^Received at least one dose of rabies vaccine.

^f^686 (57.8%) cases received Vero cell rabies vaccine, and 166 (14.0%) received hamster kidney cell rabies vaccine. 335 cases (28.2%) were missing data.

Case investigation data indicated that 1,281 (12.9%) of the total 10,971 cases experienced category I wounds. After confirming exposure routes, 727 cases were bitten or scratched by animal. However, further details regarding the wounds were lacking, and a definitive exposure category could not be confirmed, therefore, 727 cases were reclassified as having category “II or III” wounds. The remaining 554 cases could not be assigned an exposure category due to missing data or unknown exposure route, and they were defined as having an “unknown” wound. The majority of rabies-related wounds were classified as “category II” (n = 2,725; 27.4%), “category III” (n = 5,927; 59.7%), “category II or III” (n = 727; 7.3%) or “unknown” (n = 554; 5.6%). A large percentage of rabies cases (n = 4337; 40.5%) suffered bites on the head, face, neck, or hand; which are highly innervated parts of the body.

### Wound treatment, PEP vaccination, and RIG

Following rabies exposure, 1,095 (10.0%) cases sought wound treatment at a medical facility and 577 (5.3%) cases received appropriate wound treatment, including flushing and disinfection of the wound site at the medical facility. Of all cases, 1,187 (11.7%) began a rabies PEP vaccination schedule. Of these cases, the majority (n = 847; 71.4%) began the five-dose Essen regimen, 15 cases (1.3%) began the 2–1–1 Zagreb regimen, and the remaining cases vaccination schedules were missing (n = 325; 27.4%). 280 cases received RIG, more than half of whom received HRIG (n = 195; 69.6%), while 36 (12.9%) cases received ERA, data on RIG type was missing for 49 (17.5%) cases.

In bivariate analysis, sex (p < 0.01), occupation (p-values = 0.01), and age group occupation (p < 0.01) were each statistically associated with failure to begin a PEP vaccination series. There was no statistically significant difference between rural and urban cases in terms of failure to begin PEP vaccination (p = 0.36).

In the multivariable model, sex (male; adjusted odds ratios [aOR] = 1.25, 95% confidence interval [CI]: 1.09–1.44), occupation (farmer aOR = 1.39, 95% CI: 1.10–1.77) and age group (“≥55” aOR = 1.48, 95% CI: 1.01–2.17) remained statistically associated with a failure to begin PEP vaccination (Table 2).

**Table 2:** Risk factors associated with failure to begin PEP vaccination, according to data obtained from human rabies case investigations, China, 2006–2012.

Factor	Cases NOT beginning PEP vaccination	Cases Beginning vaccination	Unadjusted OR (95% CI)	Adjusted OR (95% CI)
Sex				
Female	2,359	394	Ref	Ref
Male	5,416	733	1.23 (1.08–1.41)	1.25 (1.09–1.44)
Occupation				
Others^a^	582	92	Ref	Ref
Farmer	5,748	665	1.37 (1.08–1.73)	1.39 (1.10–1.77)
Student^b^	855	198	0.68 (0.52–0.89)	0.95 (0.64–1.42)
Children^c^	376	137	0.43 (0.32–0.58)	0.63 (0.41–0.98)
Age (years)				
<15	1,168	330	Ref	Ref
15–54	3,147	393	2.26 (1.93–2.66)	1.42 (0.99–2.03)
≥55	3,291	378	2.46 (2.09–2.89)	1.48 (1.01–2.17)
Area				
Urban	1,538	205	Ref	—
Rural	5,378	774	0.93 (0.78–1.09)	—

OR = odds ratio. Ref = reference.

^a^Teachers, laborers, self-employed and unemployed, workers, food industry personnel, retired and cadres of staff, etc.

^b^Primary, secondary and college students.

^c^Children attending or not attending kindergarten.

Among cases with category II or above exposures who began PEP vaccination (n = 1,127), 224 (19.9%) cases completed the entirety of the vaccination series according to the recommended schedule (4 for the 2–1–1 Zagreb regimen and 220 for the five-dose Essen regimen) (Table 3). Of the 5,927 cases with category III exposures, 234 cases (3.9%) received RIG, but only 42 cases (0.7%) received both a complete PEP vaccination series and RIG as recommended. Of the 632 cases beginning but not completing a PEP vaccination series, 440 cases (69.6%) developed symptoms of rabies during the PEP vaccination schedule. Furthermore, 47 cases (7.4%) did not believe that finishing the series was necessary, 11 cases (1.7%) were unable to afford the remaining PEP vaccinations, 10 cases (1.6%) cases developed adverse vaccine reaction, and in one case (0.2%), the doctor did not think the case needed to complete the series. Reasons for incomplete vaccine series for the remaining 123 cases (19.5%) were missing.

**Table 3:** Completion of post-exposure prophylaxis by wound exposure category, according to data obtained from human rabies case investigations, China, 2006–2012.

Item	Exposure category, n (%)			Total, n (%)
	II	III	II or III^c^	
Wound treatment^a^	2.2% (61/2,725)	8.4% (496/5,927)	1.8% (13/727)	6.1% (570/9,379)
Vaccination^b^	1.4% (37/2,725)	3.0% (175/5,927)	1.7% (12/727)	2.4% (224/9,379)
Wound treatment^a^ + Vaccination^b^	0.6% (15/2,725)	1.3% (78/5,927)	0.4% (3/727)	1.0% (96/9,379)
Wound treatment^a^ + Vaccination^b^ + Rabies immunoglobin	0.1% (3/2,725)	0.5% (28/5,927)	0.0% (0/727)	0.3% (31/9,379)

Note: ^a^Normative wound treatment in medical institution. ^b^Complete vaccination course. ^c^Exposure caused by animal bitten or scratch but classified as category I by medical staff.

Of the 440 cases who developed symptoms of rabies before completing the vaccination schedule, 209 cases (47.5%) did not receive recommended wound treatment (wound flushing and disinfection) at a medical facility, and 116 cases (26.4%) with category III exposure or bites at rich innervation areas (head, face, neck, hand) did not receive RIG. Of these 440 cases, 173 (39.3%) received wound flushing and disinfection at a medical facility within one day of exposure and 283 (64.3%) began PEP vaccination within one day of exposure. With regard to RIG, only 89 (20.2%) cases received RIG within one day of exposure.

The majority of cases with exposure classified as category II or above received wound treatment (n = 726; 90.6%), PEP vaccination (n = 649; 76.6%), and RIG (n = 171; 79.2%) at a medical facility on the same day as exposure (Table 4). 31 cases who were clinically diagnosed with rabies received the complete PEP vaccination series and RIG, along with integrated wound treatment in the medical facility, regardless of exposure category, but still died as a result of rabies (see Supplementary Table S2**)**. Of these 31 cases, 30 (96.8%) initiated PEP vaccination within one day of exposure (see Supplementary Table S2**)**.

**Table 4:** Time interval from rabies exposure to post-exposure prophylaxis by exposure category, according to data obtained from human rabies case investigations, China, 2006–2012.

Interval	Exposure category, n (%)			Total, n (%)
	II	III	II or III^a^	
**From exposure to wound treatment in medical institution**				
Within 1 day	85 (83.3)	617 (91.5)	24 (96.0)	726 (90.6)
1 day	5 (4.9)	35 (5.2)	1 (4.0)	41 (5.1)
>1 day	12 (11.8)	22 (3.3)	0 (0.0)	34 (4.3)
Total	102 (100.0)	674 (100.0)	25 (100.0)	801 (100.0)
**From exposure to vaccination**				
Within 1 day	79 (60.8)	549 (79.2)	21 (87.5)	649 (76.6)
1 day	12 (9.3)	81 (11.7)	1 (4.2)	94 (11.1)
>1 day	39 (29.9)	63 (9.1)	2 (8.3)	104 (12.3)
Total	130 (100.0)	693 (100.0)	24 (100.0)	847 (100.0)
**From exposure to injecting RIG**				
Within 1 day	7 (46.7)	163 (81.5)	1 (100.0)	171 (79.2)
1 day	3 (20.0)	21 (10.5)	0 (0.0)	24 (11.1)
>1 day	5 (33.3)	16 (8.0)	0 (0.0)	21 (9.7)
Total	15 (100.0)	200 (100.0)	1 (100.0)	216 (100.0)

^a^Exposure caused by animal bite or scratch but classified as category I by medical staff.

### Clinical characteristics of the rabies cases

We received information on the clinical characteristics of 10,670 rabies cases. Of these cases, almost all cases (n = 10,579; 99.2%) developed furious manifestations including hydrophobia (n = 9,158; 85.8%), aerophobia (n = 9,045; 84.8%), agitation (n = 8,074; 75.7%), photophobia (n = 6,160; 57.7%), convulsions (n = 4,718; 44.2%) and mental disorders (n = 2,428; 22.8%) (Table 5). Clinically diagnosed cases and laboratory-confirmed cases had similar manifestations.

**Table 5:** Clinical manifestations of human rabies cases by diagnosis type, China, 2006–2012.

Manifestation	All cases (N = 10,670)		Clinically diagnosed cases (N = 10,522)		Laboratory-confirmed cases (N = 148)	
	Cases (n)	%	Cases (n)	%	Cases (n)	%
Hydrophobia	9,158	85.8	9,033	85.9	125	84.5
Aerophobia	9,045	84.8	8,922	84.8	123	83.1
Agitation	8,074	75.7	7,953	75.6	121	81.8
Photophobia	6,160	57.7	6,080	57.8	80	54.1
Convulsions	4,718	44.2	4,625	44.0	93	62.8
Mental disorder^a^	2,428	22.8	2,385	22.7	43	29.1

^a^Mental status alternates between an established normal baseline and progressively more severe agitation and/or depression.

The median incubation period (time from exposure to the development of symptoms) was 66 days (interquartile range [IQR] 33–167), although this median varied by exposure category and age group. The median incubation period was 80 days (IQR 37–222) for category II and 61 days (IQR 31–138) for category III (p < 0.01) (see Supplementary Tables S3 and S4). Cases with bites on the head, face, neck, or hand had shorter incubation periods (58 days [IQR 30–115]) than cases with other wounds (76 days [IQR 37–218]) (p < 0.01). Children younger than 15 years of age had the shortest incubation period (52 days [IQR 25–127]) among all age groups, regardless of exposure category (p < 0.01) (see Supplementary Tables S3 and S4). The median clinical course duration (from symptom onset to death) was 3 days (IQR 2–5) for all cases.

## Discussion

This national level analysis of human rabies case investigations provides important information on exposure history, PEP use, and clinical characteristics of human rabies cases in China^9–12^. Overall, most persons with rabies, including those with category III exposure, did not seek health care for wound treatment or PEP vaccination, with this trend particularly prominent for males, farmers and older people. Among those who began the PEP vaccination series, less than half received all the required PEP vaccination doses. Of cases that required RIG, only a low percentage of category III patients received appropriate RIG treatments. Although clinically diagnosed and laboratory-confirmed cases presented with similar symptoms, increased laboratory testing would likely avoid possible misdiagnoses^13^.

In China, the rate of pre-exposure prophylaxis vaccination for rabies is extremely low among the general population^14^. Therefore, rabies prevention largely relies on PEP following potential rabies exposure. Appropriate and timely wound treatment can reduce rabies viral loads and risk of secondary bacterial infections within bite or scratch sites. However, our study indicated that a relatively low percentage of exposed individuals sought appropriate wound treatment at medical facilities. The low proportion of patients beginning and subsequently completing the entire PEP vaccination series was a major factor in the onset of rabies in our analysis. This is due to the mechanism of protection offered by PEP. PEP vaccination stimulates the immune system to generate antibodies to protect the body from imminent infection. Therefore, completing the entire PEP vaccination schedule is crucial to ensure sufficient antibody titers are attaining to neutralize rabies virus, and prevent the onset of active rabies infections in exposed individuals. As such, those not completing full PEP schedules would be less likely to generate protective titers of antibody to prevent infection.

For cases with category III exposure, RIG is also recommended in addition to wound treatment and PEP vaccination. Following the primary dose of vaccination, it takes approximately 7 days to generate sufficient antibodies titers (above 0.5 IU/mL) to neutralise rabies virus^15^. During this period, infiltration of RIG locally within the wound site provides additional protection by blocking the systemic spread of rabies virus. However, the percentage of patients receiving RIG among those with category III exposure was small in our analysis, indicating that the lack of administration of RIG is another important risk factor for the onset of rabies.

The small percentage of cases beginning the PEP series in our study is similar to the percentages reported in other countries. For example, in India, 20.9% and 1.3% of rabies patients initiated PEP vaccination and RIG, respectively^16^. In the Philippines, only 1.7% of rabies cases received PEP^17^. In China, the percentage of patients beginning and completing PEP as recommended could be limited by three factors: the high out-of-pocket cost of rabies vaccines and RIG, access to appropriate rabies outpatient care, and community levels of knowledge regarding rabies prevention.

According to the National Bureau of Statistics of China, the average annual income for residents of rural areas was approximately 5,490 RMB (822 US dollar) between 2006 and 2012. The average total of a complete PEP vaccination (5 doses) and RIG were more than 300 RMB (45 US dollars) and 1,000 RMB (150 US dollars), respectively. As theses cost are only partially subsidised by the national health insurance system, PEP and RIG can be prohibitively expensive for many Chinese residents, especially for farmers who were indicated in this study as high-risk individuals for rabies.

However, not all PEP vaccination schedules pose similar upfront on consequential costs. For example, compared with the five-dose Essen regimen, the Zagreb regimen with 4 vaccine doses and 3 clinic-visits can reduce direct out-of-pocket vaccination and travel expenses, and minimize lost working time, which may effectively promote PEP vaccination compliance^18,19^. Furthermore, the 3-clinic-visit intradermal PEP schedule recommended by the WHO in 2018 is even more economical than the Essen or Zagreb regimens^20^. Therefore, we advise expanding the use of the currently used Zagreb regimen in China, and additionally exploring the feasibility of alternative intradermal 3 clinic-visit vaccination schedules for rabies PEP in China.

Similarly to previous studies of human rabies in China, we found that rabies disproportionately affects those in rural areas^5^. These epidemiological trends likely reflect the unequal distribution of medical resources in China, and the logistical challenges of long distances and limited transportation to medical facilities for rural and remote mountainous communities^21,22^. Access to rabies clinics on a national scale has never been assessed, and an accurate understanding of the situation would provide evidence for a rational approach for the distribution of PEP clinics in the future. In addition, health education, bite prevention and rabies awareness is insufficient in rural areas and remote mountainous communities^23^, which may lead to reduced healthcare seeking behavior for PEP treatment due to lack of perceived risk or lack of knowledge regarding rabies prevention. The results of this study suggest that males, farmers and older age groups are at highest risk for rabies infection, and as such, should be prioritized for rabies awareness, bite reduction and infection prevention education.

Our analysis also indicated inappropriate PEP practice conducted within medical facilities. Standard wound treatment regimen includes wound flushing and disinfection, as well as surgical procedures if necessary^4,6^. Flushing and disinfection are essential steps to reduce rabies viral load and the risk of secondary bacterial infection within the wound site. However, only 52.7% of the cases treated at medical facilities received appropriate wound care including flushing and disinfection.

Additionally, according to the position of WHO and national guidelines in China, category I exposures do not lead to active rabies infection^15,20^. Our original dataset contained 1,281 cases initially classified as having category I exposure by healthcare workers. Based on the exposure routes of these wounds and our standardized classification system of rabies exposure, 727 (56.7%) of these cases should have been classified as having category II or III exposure. Because our study is retrospective in nature, we cannot confirm the exposure categories of the other 554 rabies cases (1.3% laboratory-confirmed and 98.7% clinically diagnosed) whose exposure routes were missing, these 554 cases’ exposure categories were unknown. These wounds might be very small and ignored in clinical practice, then lead to the misclassification of exposure category. However, clinicians can distinguish these small wounds by scrubbing exposed patient’s skin with alcohol wipes in clinics^15^. Therefore, the improvement of clinical practice in rabies PEP clinics is needed, especially, the classification of rabies exposure, which should be addressed in rabies intervention programmes.

The low percentage of cases with category III wounds receiving appropriate RIG in our study is also of concern, and underlying issues of appropriate care needs to be addressed. Therefore, we suggest that medical facilities require an audit of rabies PEP practices and procedures, to identify opportunities to improve healthcare worker training and awareness in order to support future rabies intervention programmes.

However, even when appropriate and timely treatment was administered regardless of exposure category, including wound flushing and disinfection, completion of a PEP vaccination series, and RIG, 31 cases in our study developed active rabies infections. Even though all 31 cases were clinically diagnosed, definitions of clinical diagnosis are strict with regards to clinical manifestation, and our analysis showed 99% cases developed symptoms of furious rabies. We believe that the most likely cause of death was rabies, although we cannot exclude the possibility that a minority of them died of other causes. These deaths could also be due to PEP failure due to non-standard practice, insufficient RIG dosing, ineffective vaccine or immunocompromised patients. To identify specific causes for PEP failure is beyond the scope of our study, but further work is required to identify potential areas for improving PEP success rates. Investigations could include the effects of source and cold-chain maintenance on vaccine efficacy, determining best-practices for identifying and appropriately monitoring antibody responses in immune deficient patients, and as previously mentioned, compliance with recommended RIG dosing in current medical facilities. We suggest assessing antibody levels after PEP vaccination for patients with documented immunodeficiency, and a booster should be administered if antibody levels drop below 0.5 IU /mL^4^.

For cases included in this study, the incubation period ranged from 1 month to 5 months after exposure, which is considerably longer than indicated in other studies, with ranges from 1 month to 2 months, and from 3 weeks to 3 months^6,13,24^. Incubation periods varied according to exposure category, wound type and age group. Category III exposures indicates that the wound is large and deep, resulting in a high viral inoculation loads and subsequently shorter incubation periods^6^. As the rabies virus is neurotropic, bites in the highly enervated areas of the head, face, neck or hands leads to shorter incubation periods^6,13^. Children younger than 15 years of age had shorter incubation periods than all other age groups. This could be due to their close interactions with pets (e.g., dogs and cats) while playing with, petting or kissing the animal, leading to higher rates of bites on the head, face, neck, or hand.

Previous studies have suggested that easily clinically distinguishable furious rabies accounts for 66% of all classic rabies cases in general. However, the remaining 33% of cases present as paralytic rabies, whose clinical manifestations are easily misdiagnosed as other diseases, in the absence of laboratory confirmation^24^. In our study, nearly all of included cases (99%) manifested as furious rabies. Because paralytic rabies has similar manifestations with myelitis and Guillain-Barre syndrome (GBS), and laboratory confirmation rates for rabies is low, patients presenting with paralytic rabies might not be correctly diagnosed by clinicians in China^25–27^. Likely misdiagnosis can lead to an underestimation of the disease burden of rabies in China. Therefore, awareness of symptomology of rabies in clinicians should be improved, so that in the case of a patient presenting with myelitis and/or GBS, clinicians will consider an alternative diagnosis of rabies. In such cases, clinicians should investigate the patient history for the possibility of exposure to a rabid animal bite or scratch, including the often overlooked exposure to bats^28^.

During the study period, dogs were the main source of human rabies in China, with more than half of cases resulting from bites from domesticated dogs. This reflects similar findings in other countries, including India^16^ and Indonesia^29^, where, like China, universal dog rabies vaccination programmes are not in affect. Currently in China, dogs are required to be registered and receive an annual examination, which could cost between 500RMB and 1,000 RMB (75–150 US dollars) in Beijing^30^. Although dog rabies vaccinations are offered for free in some developed cities (e.g., Hangzhou, Shenzhen), even for unlicensed dogs, in most areas, owners must pay for dog rabies vaccinations annually, costing between 50–80 RMB (7–12 US dollars)^31^. However, due to the high cost and lack of awareness of dog vaccinations, limited numbers of trained veterinarians, and the existence of a large number of free-roaming dogs, dog vaccination coverage remains far below the 70% needed to interrupt rabies transmission in dogs^7,32–35^.

WHO indicates that rabies virus infection in rodents is very uncommon; similarly, 26 cases (0.2%) reported exposure to rodents in our analysis. These cases were clinically diagnosed cases who presented with typical clinical manifestations but were not laboratory confirmed. As such, we could not confirm that rodents caused these human rabies cases. However, rabies virus infection in rodents has been detected by laboratory testing in both the United States and China, and our results suggest that it is possible that exposure to rodents may cause human rabies^20,36,37^. Further investigation to confirm this transmission route requires confirmation by a detailed case reporting, alongside laboratory evidence in the future.

Our study had several limitations. We collected information on only 66% of all human rabies cases reported through the NIDRIS in China from 2006 to 2012. As such, we were unable to assess whether the exposure characteristics and clinical courses of the cases included in this analysis were representative of all cases. Additionally, if patients died before the case-investigation was completed, or the patient’s health was too compromised to complete the case-investigation, information was obtained from relatives, and therefore certain details about exposure characteristics and PEP were unavailable. Nevertheless, our large sample size and analysis provide critical information for rabies programme directors at the national and local levels in China.

Based on the recommendations of the WHO and experiences of other countries, an integrated intervention strategy including mass dog vaccinations, providing access to human rabies PEP and enhancing community awareness of rabies prevention methods and programmes, is efficacious for the control and elimination of dog-mediated human rabies^38^. Preventing and controlling rabies has been highlighted in the Long-Term Animal Disease Prevention and Control Plan (2012–2020), issued by the Chinese State Council as a guidance document for related departments. To achieve the goal of controlling and eliminating rabies in China, an integrated intervention strategy modelled on WHO recommendations should be adopted, which will require the collective efforts of multiple sectors, including public health departments, veterinary departments, city management offices, and education departments^39^.

Despite current national policy guidance in China, most cases did not begin or complete a PEP vaccination series. As such, improving adherence to PEP practices at health care facilities that treat bite wounds from animals should be addressed by rabies intervention campaigns. Additionally, based on our findings, to eliminate dog-mediated human rabies, rabies prevention campaigns targeting males, farmers and person older than 55 years should be conducted alongside general population campaigns. Since dogs, including domesticated dogs, are the primary cause of human rabies, additional approaches to increasing dog vaccinations should also be explored. Finally, in order to strengthen surveillance efforts, and accurately assess the burden of rabies and effectively monitor the impact of rabies intervention programmes, rates of laboratory-confirmation of suspected human rabies cases should be improved.

## Methods

### Data collection

In 2013, we administered a standard questionnaire to all 31 provinces in mainland China to collect information regarding investigations of human rabies cases with dates of onset between January 1, 2006, and December 31, 2012. The questionnaire collected data on the following: date of exposure, location of exposure, exposure route, exposure category, anatomical site of wound, vaccination history of the animal involved, the patient’s clinical characteristics, wound treatment (time, medical institution, operating steps), PEP vaccination (time, date, kind of vaccine and vaccination series), reasons for failing to complete the PEP vaccination schedule and receipt of RIG. Provincial staff treating rabies were asked to collect paper-based questionnaires in all case investigations, enter relevant data into an EpiData 3.1 dataset, and return the electronic dataset to China CDC.

### Data management and analysis

Case-investigation data were imported into SAS 9.3 (Cary, NC, USA) for cleaning and analysis. We removed duplicate records and cases with onset dates outside of the 2006 to 2012 project period. We cross-referenced investigation data with rabies case reported to the NIDRIS; cases that were previously reported to the national surveillance system were retained for our analysis (see Supplementary Fig. S1). Missing data from each record were categorized as “missing”.

Before 2008, the criteria of the rabies diagnosis were unified in the National Rabies Surveillance Program published by the Chinese Ministry of Health in 2005. The criteria indicate human rabies cases were classified as clinically diagnosed cases if a patient had been licked, bitten or scratched by dog, cat or other mammal, with clinical symptoms of a prickling or itching sensation at the site of the bite, progressing within days to agitation, anxiety, confusion, hydrophobia, aerophobia, and paralysis of muscles or cranial nerves. Alternatively, human rabies cases were classified as laboratory-confirmed cases if a person had a clinical diagnosis of rabies and any one of the following: laboratory evidence of rabies infection detected by direct fluorescent antibody test (DFA), reverse-transcriptase polymerase chain reaction (RT-PCR) or rabies virus isolation testing in clinical specimens.

In 2008, these criteria were modified and published as the *Standard of Rabies Diagnosis (WS 281-2008)* by the National Health and Family Planning Commission of the People’s Republic of China^40^. Rabies was classified as furious rabies or paralytic rabies based on clinical symptoms. The clinical symptoms of furious rabies were similar to those defined in the criteria before 2008. However, in paralytic rabies, which lacks hyperactivity or hydrophobia, muscles gradually become paralyzed, starting at the site of the bite or scratch, and progress with systemic flaccid paralysis. A clinically diagnosed case of rabies was defined as the occurrence of typical manifestations in a patient with a history of exposure to animals with rabies^5^. Laboratory-confirmed cases were defined as clinically diagnosed cases with any one of the following: laboratory evidence of rabies infection detected by DFA, RT-PCR or rabies virus isolation testing in clinical specimens. Medical staff categorized wounds according to increasing severity as follows:

Category I - touching or feeding animals, licks on intact skin;

Category II - nibbling on uncovered skin or minor scratches or abrasions without bleeding;

Category III - single or multiple transdermal bites or scratches, licks on broken skin, and contamination of mucous membrane with saliva from licks^6^.

We verified the variable “Exposure route” to check the exposure category of wound classified by medical staff. Exposures caused by animal bites or scratches but classified as category I by medical staff were reclassified as category “II or III”. Exposures classified as category I by medical staff, but the exposure route was unknown or missing were reclassified as “unknown”. Exposures classified as category II or category III by medical staff were not reclassified (See Supplement Table S1**)**.

We described the demographic characteristics, exposures history, and clinical characteristics of rabies cases as well as the timing and type of PEP initiated. Using cases in exposure category II or above, we performed logistic regression analysis to identify factors associated with failing to begin an appropriate PEP vaccination series. Our independent variables included sex, occupation, area (rural vs. urban) and age group. The dependent variable was dichotomized as a failing to initiate vaccination – yes or no. Odds ratios (ORs) and 95% confidence intervals (CIs) were calculated. Variables statistically significant in the crude univariable analyses were included in the multivariable model.

We also assessed differences in incubation period according to case definition (clinically diagnosed or laboratory-confirmed), exposure category (category II or above), the type of wound (sensitive or other wounds) and age group using T-tests and analysis of variance (general linear model [GLM]). We described the clinical course of infection and outlined reasons for failing to complete the full PEP vaccination series if initiated. An alpha level of 0.05 was used to assess statistical significance.

### Ethical approval

Data analysed in this project were obtained through ongoing public health surveillance of a notifiable infectious disease. The National Health and Family Planning Commission, China, determined that the investigation of human rabies cases was part of a continuing public health surveillance activity and was exempt from institutional ethical review board assessment. The project was also determined to be a routine public health surveillance activity, in accordance with human subjects’ protection procedures of the United States CDC (CGH #2015-238). All analysed data were anonymized and thus did not include any personal identifying information.

## Electronic supplementary material

Supplementary information

## Data Availability

The data that support the findings of this study are available from China CDC but restrictions apply to the availability of these data, which were used under license for the current study, and so are not publicly available. Data are, however, available from the authors upon reasonable request and with permission of China CDC.
