# Supplementary material for: Exposure history, post-exposure prophylaxis use, and clinical characteristics of human rabies cases in China, 2006–2012
Source: Sci Rep. 2018 Nov 21;8:17188. doi: 10.1038/s41598-018-35158-0 (PMC6249250; doi:10.1038/s41598-018-35158-0)
Supplement: Supplementary file 1 — Supplementary information [file 41598_2018_35158_MOESM1_ESM.docx]

**Supplementary Information**

**Exposure history, post-exposure prophylaxis use, and clinical characteristics of human rabies cases in China, 2006-2012**

**Author list：**

Chun Guo, Yu Li, Yang Huai, Carol Y. Rao, Shengjie Lai, Di Mu, Wenwu Yin , Hongjie Yu, Shaofa Nie

**Supplementary Figure S1. Procedure for selecting the questionnaire records of clinically diagnosed or laboratory-confirmed rabies cases in China between January 1, 2006, and December 31, 2012**

12501 questionnaire records

12201 questionnaire records were retained after de-duplicating

10971 questionnaire records included in this study

300 duplicated questionnaire records

931 questionnaire records belonged to suspected rabies case

299 records belonged to cases whose onset times were not between 2006/1/1 and 2012/12/31

11902 records were retained

16628 clinical diagnosed rabies cases or laboratory-confirmed rabies cases in NIDRIS between whose onset times were 2006/1/1 and 2012/12/31

Details about the procedure:

1. Checked the duplicate collected records, if records conformed to any of the following conditions;
2. The same name (including the same pronunciation name), the same sex, the same age;
3. The same name (including the same pronunciation name), missing sex, the same age, similar residential address and onset date;
4. The same name (including the same pronunciation name), missing sex, an age difference of less than 2 years old, similar residential address and onset date;
5. Deleting duplicate records; the record which conformed to any of the following conditions was preserved:
6. Had earlier onset date;
7. If both records had the same onset date, record containing the death date was preserved;
8. If both records had the same onset date and death date, the record with least missing data was preserved.
9. Because very few local CDCs also report cases in 2005 and 2013 and were outside of the study timeframe, these cases were not included.
10. Linked the clinically diagnosed rabies cases and laboratory-confirmed rabies cases from NIDRIS with the questionnaire records. If case’s basic information in questionnaire record was similar to cases from NIDRIS (referred to the conditions showed in duplicate checking step), they would be linked successfully. The records of suspected rabies cases would fail to be linked.

| **Supplement Table S 1. The exposure route by exposure category classified by medical staff prior to reclassification for this study** | | | | | |
| --- | --- | --- | --- | --- | --- |
| **Exposure route** | | **Category classified by medical staff**  **N (%)** | | | **Total**  **N (%)** |
|  |  | **I** | **II** | **III** |  |
| Bite | 613 (47.8) | | 2,034 (74.6) | 5,131 (86.6) | 7,778 (78.3) |
| Scratch | 114 (8.9) | | 325 (11.9) | 234 (3.9) | 673 (6.8) |
| Unknown | 61 (4.8) | | 37 (1.4) | 57 (1.0) | 155 (1.6) |
| Missing | 493 (38.5) | | 329 (12.1) | 505 (8.5) | 1,327 (13.3) |
| Total | 12,81 (100.0) | | 2,725 (100.0) | 5,927 (100.0) | 9,933 (100.0) |

| **Supplementary Table S 2. The exposure characteristics, PEP treatments and clinical features of 31 cases who, despite completing PEP developed active rabies infection** | | | |
| --- | --- | --- | --- |
| **Characteristic** | **Exposure category**  **n (%)** | | **Total**  **n (%)** |
|  | **II** | **III** |  |
| Diagnosis type |  |  |  |
| Clinically diagnosed case | 3 (100.0) | 28 (100.0) | 31 (100.0) |
| Laboratory-confirmed case | 0 (0.0) | 0 (0.0) | 0 (0.0) |
| Total | 3 (100.0) | 28 (100.0) | 31 (100.0) |
| Province |  |  |  |
| Anhui | 0 (0.0) | 1 (3.2) | 1 (3.2) |
| Guangxi | 0 (0.0) | 7 (22.6) | 7 (22.6) |
| Guizhou | 0 (0.0) | 2 (6.5) | 2 (6.5) |
| Hebei | 0 (0.0) | 3 (9.7) | 3 (9.7) |
| Hubei | 0 (0.0) | 2 (6.5) | 2 (6.5) |
| Hunan | 2 (66.7) | 5 (16.7) | 7 (22.6) |
| Jiangsu | 1 (33.3) | 3 (9.7) | 4 (12.8) |
| Shandong | 0 (0.0) | 1 (3.2) | 1 (3.2) |
| Sichuan | 0 (0.0) | 3 (9.7) | 3 (9.7) |
| Chongqing | 0 (0.0) | 1 (3.2) | 1 (3.2) |
| Total | 3 (100.0) | 28 (100.0) | 31 (100.0) |
| Year |  |  |  |
| 2007 | 2 (66.7) | 7 (25.0) | 9 (29.0) |
| 2008 | 0 (0.0) | 8 (28.6) | 8 (25.8) |
| 2009 | 0 (0.0) | 7 (25.0) | 7 (22.6) |
| 2010 | 0 (0.0) | 1 (3.6) | 1 (3.2) |
| 2011 | 1 (33.3) | 1 (3.6) | 2 (6.5) |
| 2012 | 0 (0.0) | 4 (14.2) | 4 (12.9) |
| Total | 3 (100.0) | 28 (100.0) | 31 (100.0) |
| Area |  |  |  |
| Urban | 1 (33.3) | 2 (7.1) | 3 (9.7) |
| Rural | 1 (33.3) | 22 (78.6) | 23 (74.2) |
| Missing | 1 (33.3) | 4 (14.3) | 5 (16.1) |
| Total | 3 (100.0) | 28 (100.0) | 31 (100.0) |
| Exposure Route |  |  |  |
| Bite | 3 (100.0) | 28 (100.0) | 31 (100.0) |
| Scratch | 0 (0.0) | 0 (0.0) | 0 (0.0) |
| Total | 3 (100.0) | 28 (100.0) | 31 (100.0) |
| Full vaccination series ^a^ |  |  |  |
| Yes | 3 (100.0) | 28 (100.0) | 31 (100.0) |
| No | 0 (0.0) | 0 (0.0) | 0 (0.0) |
| Total | 3 (100.0) | 28 (100.0) | 31 (100.0) |
| Time from exposure to wound treatment |  |  |  |
| Within 1 day | 2 (66.7) | 28 (100.0) | 30 (96.8) |
| Missing | 1 (33.3) | 0 (0.0) | 1 (3.2) |
| Total | 3 (100.0) | 28 (100.0) | 31 (100.0) |
| Time from exposure to vaccination |  |  |  |
| Within 1 day | 2 (66.7) | 28 (100.0) | 30 (96.8) |
| Missing | 1 (33.3) | 0 (0.0) | 1 (3.2) |
| Total | 3 (100.0) | 28 (100.0) | 31 (100.0) |
| Time from exposure to injecting RIG |  |  |  |
| Within 1 day | 0 (0.0) | 25 (89.3) | 25 (80.6) |
| 1 day | 1 (33.3) | 2 (7.1) | 3 (9.7) |
| 2 days | 0 (0.0) | 1 (3.6) | 1 (3.2) |
| Missing | 2 (66.7) | 0 (0.0) | 2 (6.5) |
| Total | 3 (100.0) | 28 (100.0) | 31 (100.0) |
| Incubation period |  |  |  |
| <3 months | 0 (0.0) | 20 (71.4) | 20 (64.5) |
| 3-13 months | 1(33.3) | 8 (28.6) | 9 (29.0) |
| Missing | 2 (66.7) | 0 (0.0) | 2 (6.5) |
| Total | 3 (100.0) | 28 (100.0) | 31 (100.0) |
| 1. 30 case-patients used 5-doses Essen regimen and received at least 5 doses of rabies vaccine, 1 case-patient used Zagreb regimen and received 4 doses of rabies vaccine. | | | |

| **Supplementary Table S3. The incubation period of rabies cases according to exposure category, wound type, and age group in China, 2006–2012** | | | | | | | | | |
| --- | --- | --- | --- | --- | --- | --- | --- | --- | --- |
| **Interval** |  |  | **Median** | **P_25_** | **P_75_** | **IQR** | ***t/F*** | | **p-value** |
| Incubation period (day) |  |  | 66 | 33 | 167 | 134 |  | |  |
|  | Diagnosis type | Clinically diagnosed case | 66 | 33 | 168 | 135 | -0.2 | | 0.85 |
|  |  | Laboratory-confirmed case | 64.5 | 33 | 134 | 101 |  |  |  |
|  |  | II | 80 | 37 | 222 | 185 |  | |  |
|  | Exposure category | III | 61 | 31 | 138 | 107 | 16.4 | | <0.01 |
|  |  | II or III ^a^ | 83 | 42 | 194 | 152 |  |  |  |
|  | The type of wound | Sensitive wound ^b^ | 58 | 30 | 115 | 85 | 11.4 | <0.01 | |
|  |  | Other wounds | 76 | 37 | 218 | 181 |  |  |  |
|  | Age group (years) | <15 | 52 | 25 | 127 | 102 | 19.8 | | <0.01 |
|  |  | 15-54 | 72 | 35 | 203 | 168 |  |  |  |
|  |  | ≥55 | 68 | 35 | 158 | 123 |  |  |  |
| Note: a. Exposure caused by animal bitten or scratch but classified as category I by medical staff. b. bite on head, face, neck, or hand, which are highly innervated parts of body | | | | | | | | | |

| **Supplementary Table S4 The result of multivariate analysis of variance of incubation period** | | | | | |
| --- | --- | --- | --- | --- | --- |
| Source | DF | Type III SS | MS | *F* | *P* |
| Age group | 2 | 21716574.5 | 10858287.3 | 5.1 | <0.01 |
| Exposure category | 2 | 24211519.0 | 12105759.5 | 5.6 | <0.01 |
| The type of wound | 1 | 67425943.6 | 67425943.6 | 31.4 | <0.01 |
| Age group* Exposure category | 4 | 14580308.8 | 3645077.2 | 1.7 | 0.15 |
| Age group*the type of wound | 2 | 28422120.8 | 14211060.4 | 6.6 | <0.01 |
| Exposure category*the type of wound | 2 | 1225189.0 | 612594.5 | 0.3 | 0.75 |
